# Supplementary material for: Development of chimeric peptides to facilitate the neutralisation of lipopolysaccharides during bactericidal targeting of multidrug-resistant Escherichia coli
Source: Commun Biol. 2020 Jan 23;3:41. doi: 10.1038/s42003-020-0761-3 (PMC6978316; doi:10.1038/s42003-020-0761-3)
Supplement: Supplementary file 1 — Supplementary Information [file 42003_2020_761_MOESM1_ESM.pdf]

Supplementary information

Supplementary Figures

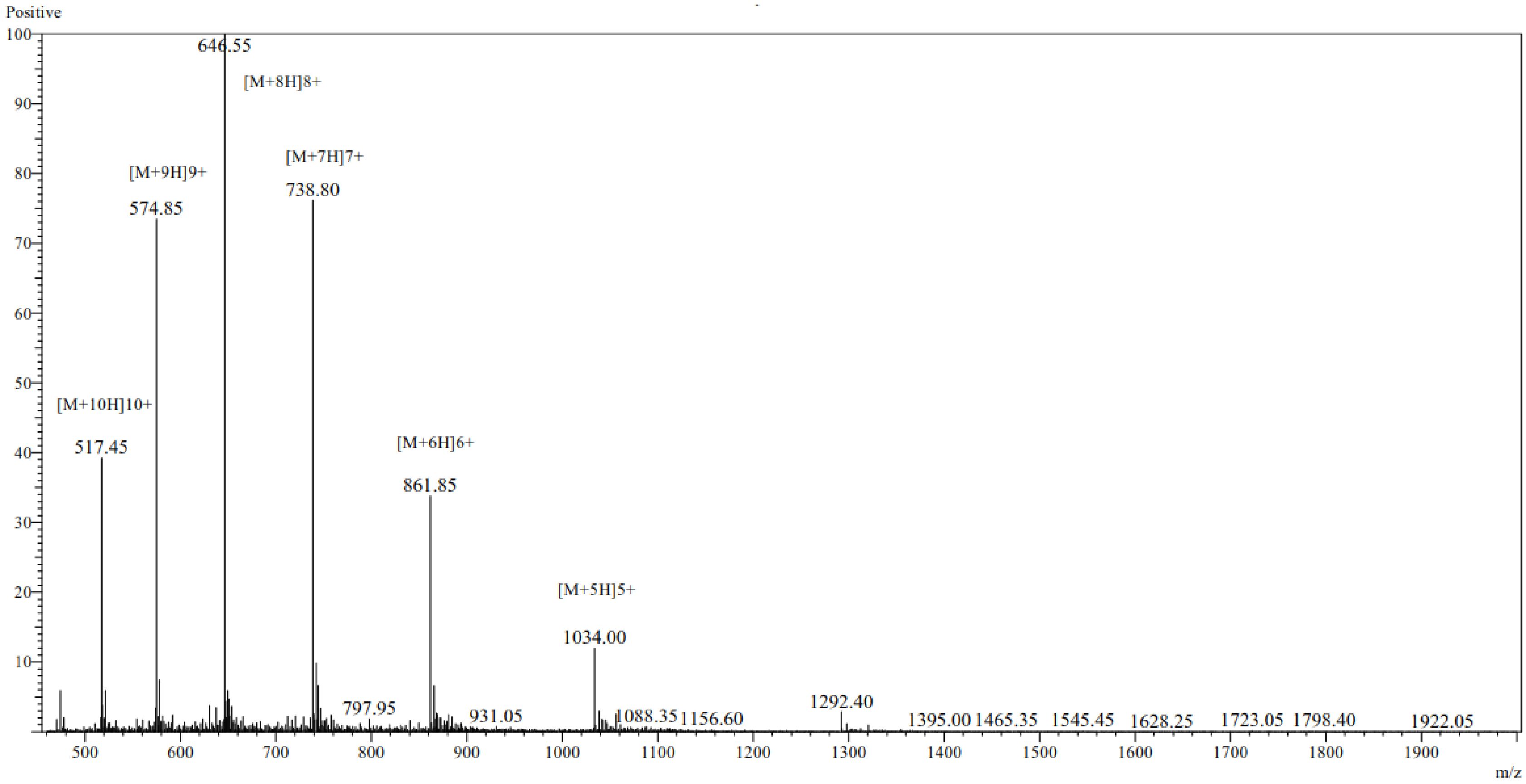

Supplementary Figure 1. ESI-MS analysis of the purified A6.

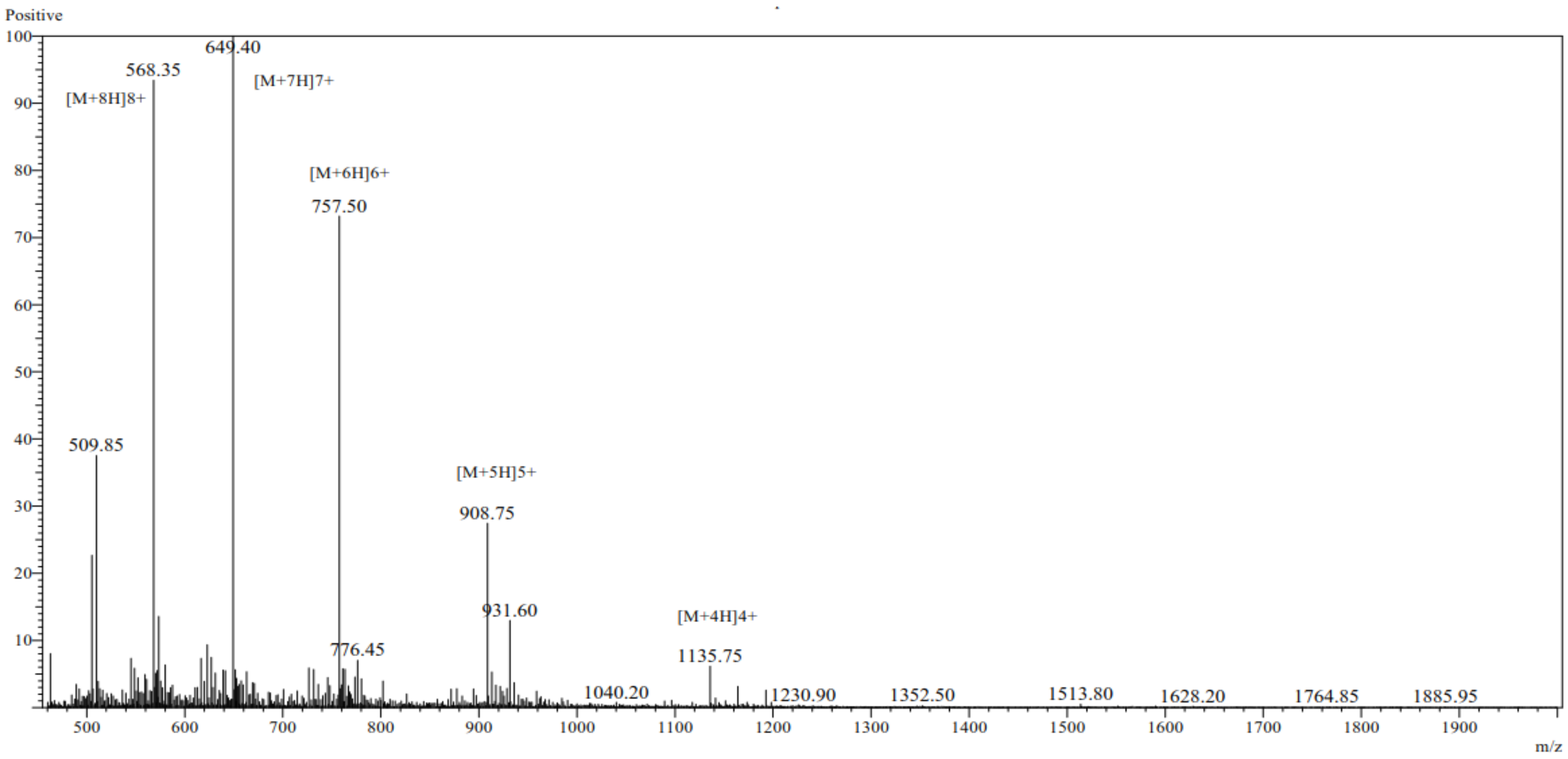

Supplementary Figure 2. ESI-MS analysis of the purified G6.

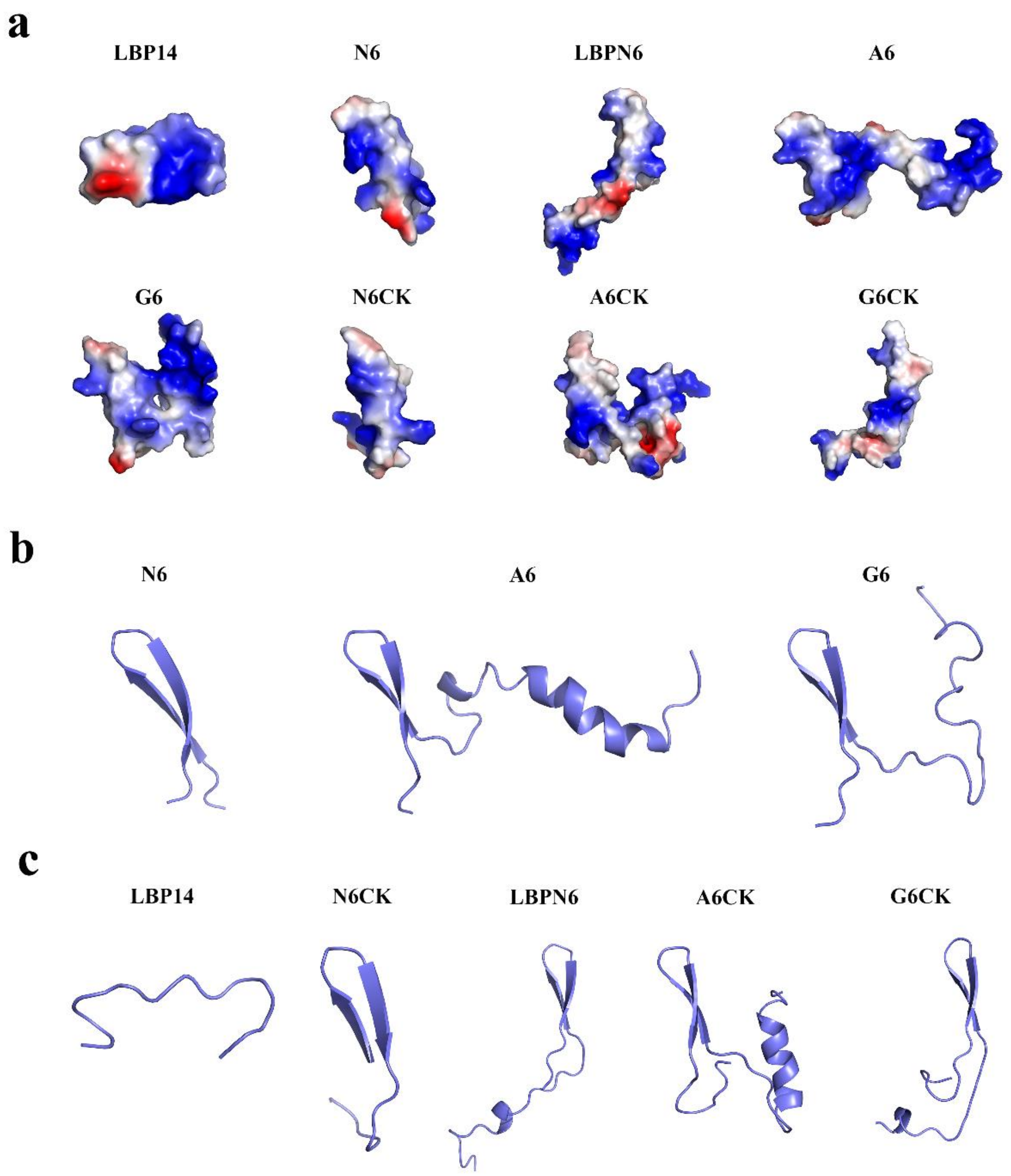

Supplementary Figure 3. Structural analysis of peptides. a Electrostatic potential surface of SCPs-A6, G6 and N6. Blue, red and white represent positive, negative and neutral charge, respectively. Molecular models were generated with PyMOL 1.8. **b** NMR analysis of A6, G6 and N6 in aqueous solutions. **c** Structures of scramble peptides analyzed by I-TASSER (Version 5.1) and Phyre (Version 2.0). LBP14N6 without linkers was used as a control; N6CK, A6CK and G6CK were designed as scramble controls of N6, A6, and G6, respectively.

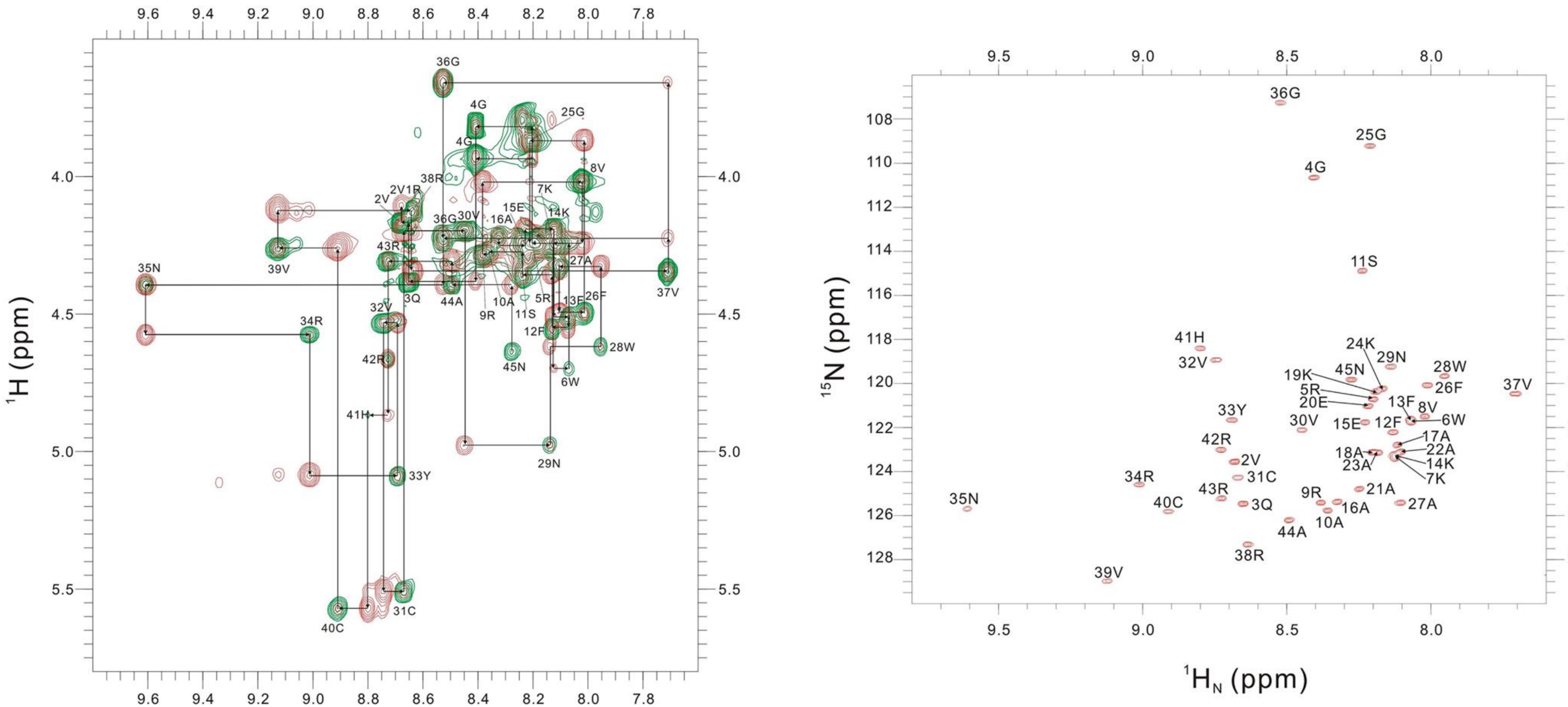

Supplementary Figure 4. The finger print region of (left) and part of the 1H-15N HSQC spectrum (right) of A6. In the finger print region, the TOCSY (green) and NOESY (red) spectra were overlaid and the connectivity of the residues was indicated with arrowed lines.

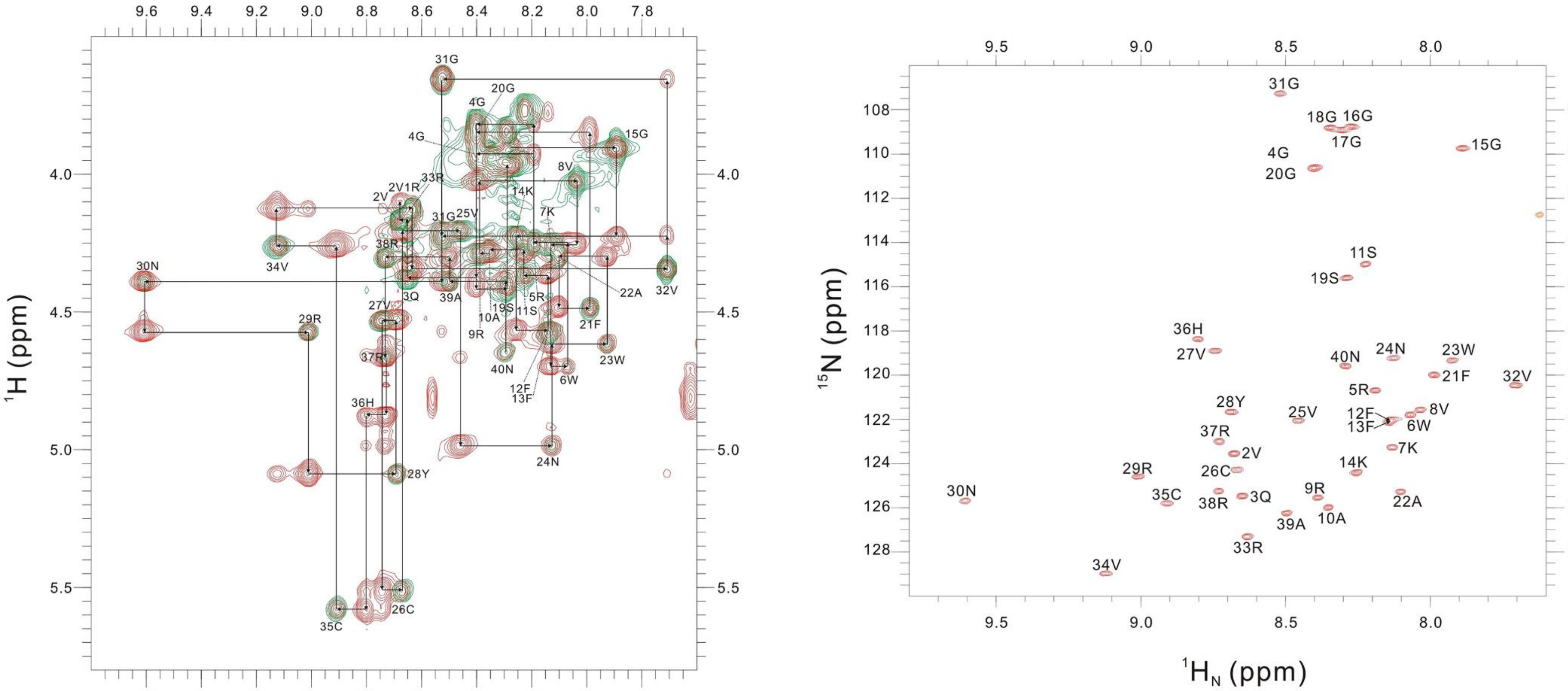

Supplementary Figure 5. The finger print region of (left) and part of the 1H-15N HSQC spectrum (right) of G6. The two has the same X axis. In the finger print region, the TOCSY (green) and NOESY (red) spectra were overlaid and the connectivity of the residues was indicated with arrowed lines. The cross-peaks of the intra-residue HN-Hα were labeled for clarity.

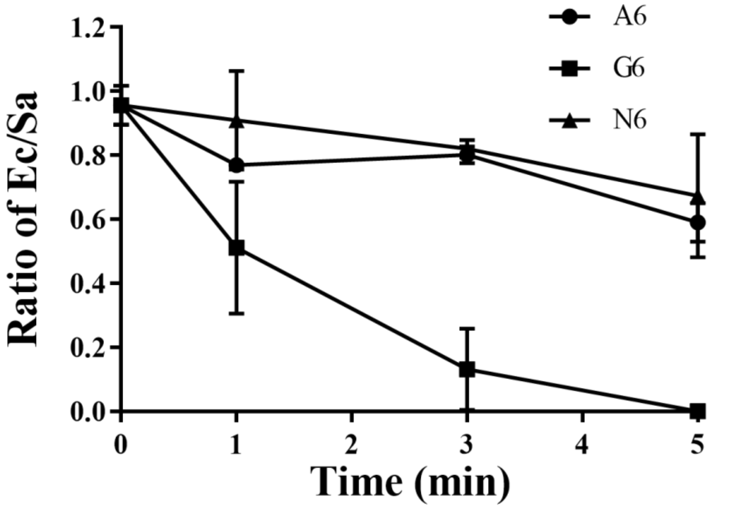

Supplementary Figure 6. Killing selectivity of peptides. A 1:1 mixture of *E. coli* (Ec) and *S. aureus* (Sa) was treated with A6 (16 μg per ml), G6 (16 μg per ml) or N6 (2 μg per ml) for 1, 3 and 5 min, respectively; survivors were counted in MH plates. The change in relative ratio of *E. coli* to *S. aureus* (Ec/Sa) after treatment with A6, G6 or N6 is shown. Results indicate means with SD (n = 3 independent experiments).

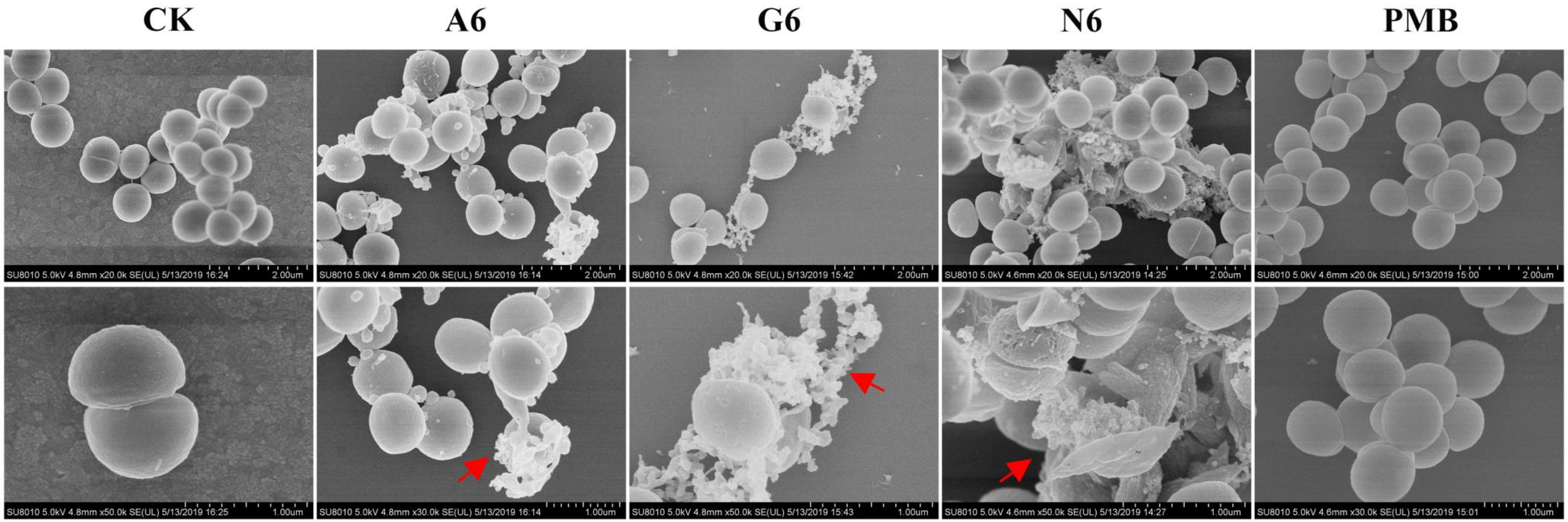

Supplementary Figure 7. Effects of SCPs-A6 and G6 on the cell morphology and ultrastructure of MDR *S. aureus* CVCC43300. Bacteria in mid-logarithmic growth were treated with peptides or antibiotic at 4 × MIC for 2 h. Red arrows indicated typical disruptions, which was caused by peptides (blebs, leakage of contents, and sheets) or PMB. The scalebar represents 2 and 1 μm.

36

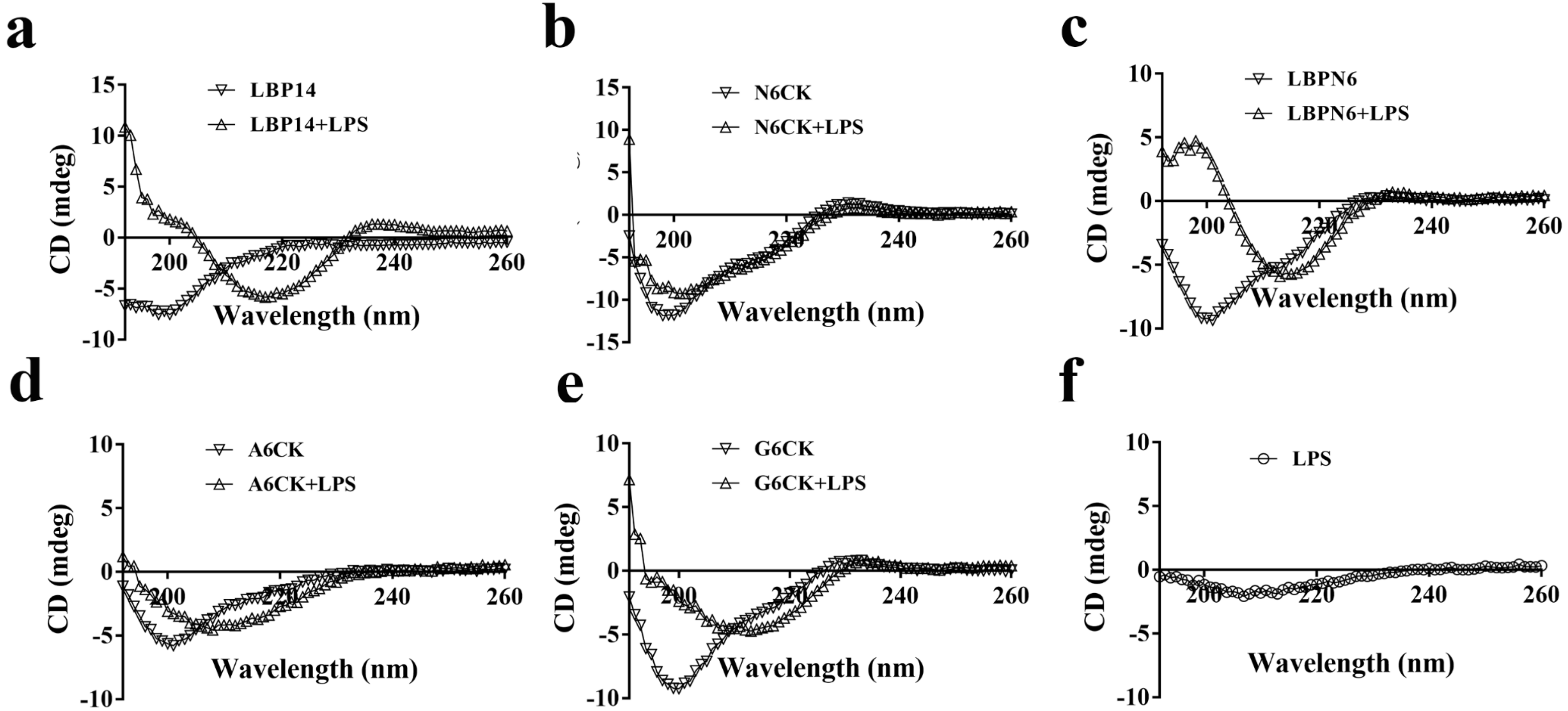

37 **Supplementary Figure 8. CD spectra for peptides with or without *E. coli* LPS (0.2 mg per ml). a** LBP. **b** N6CK. **c** LBPN6. **d** A6CK. **e** G6CK. **f** LPS.

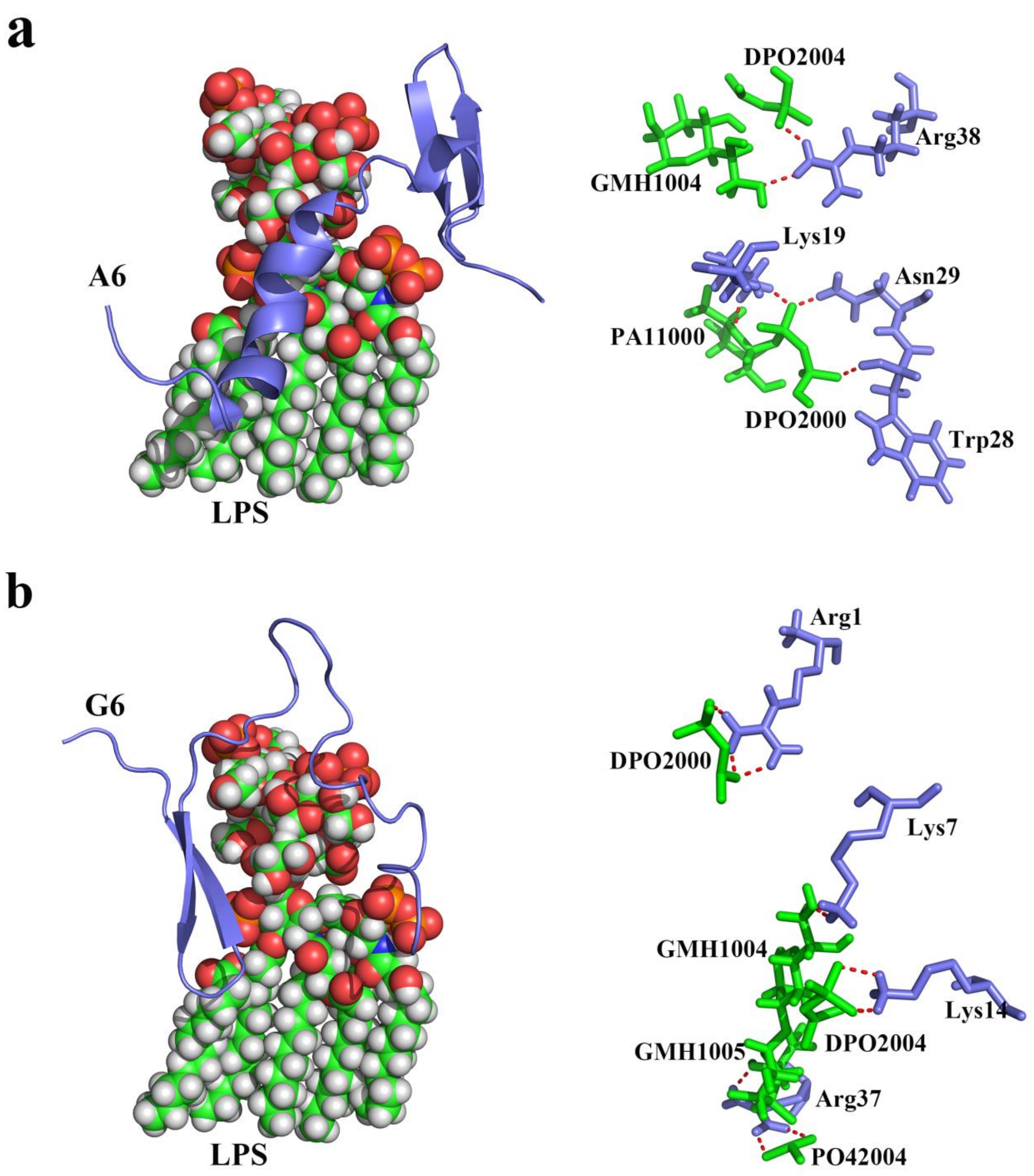

Supplementary Figure 9. Molecular docking (MD) of peptides and LPS interaction. Docking was performed using Autodock4.2. Left: complex structures of A6 **(a)** or G6 **(b)** and LPS. Oxygen, carbon, hydrogen atoms are indicated as red, green and white, respectively. Right: the residues participating in hydrogen bonding in peptides. The receptor (LPS) and peptide chains of A6 or G6 are shown as green and blue, respectively. The hydrogen bonds are indicated as red dotted lines.

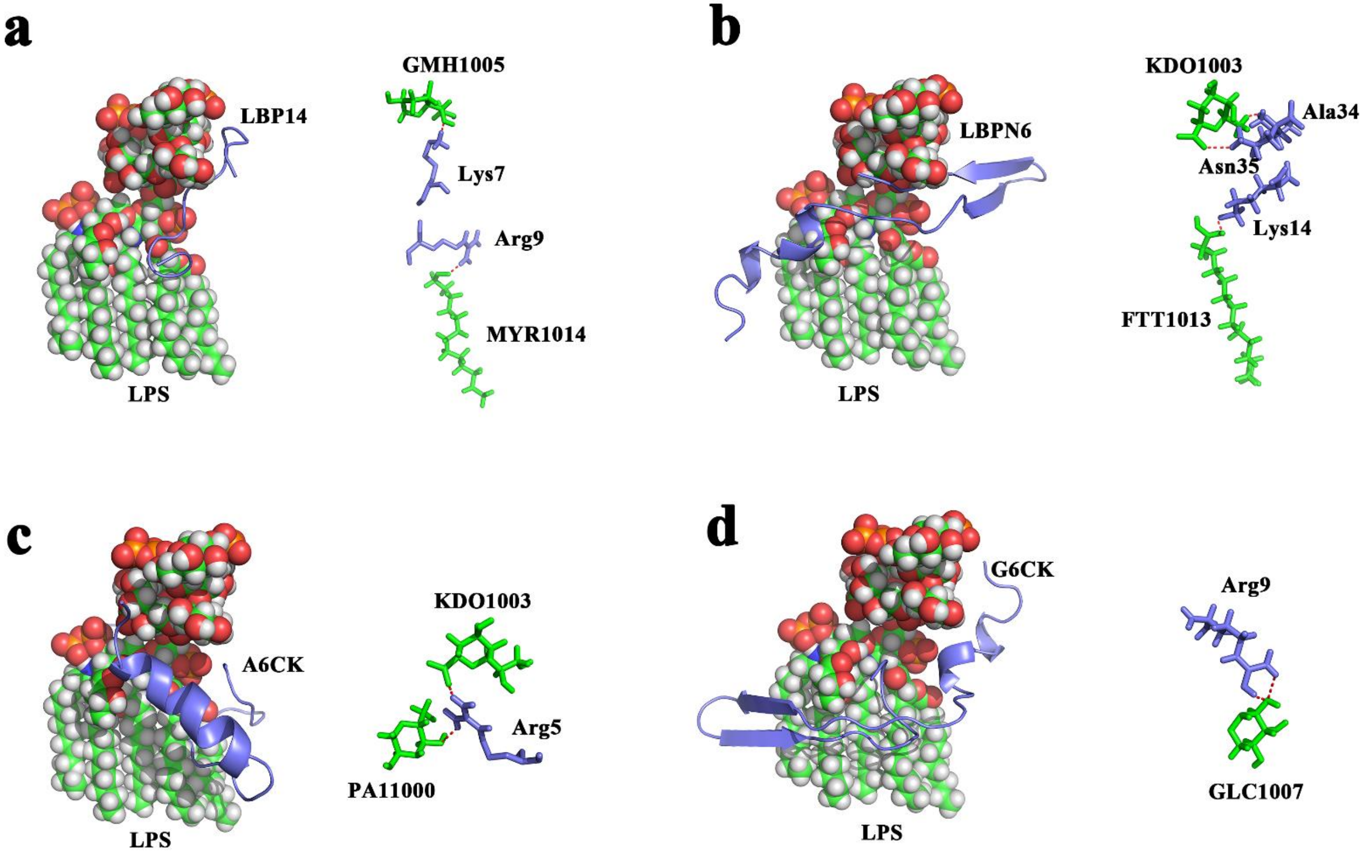

Supplementary Figure 10. Molecular docking (MD) of peptides and LPS interaction. Docking was performed using Autodock4.2. Left: complex structures of LBP14 **(a)**, LBPN6 **(b)**, A6CK **(c)** or G6CK **(d)** and LPS. Oxygen, carbon, hydrogen atoms are indicated as red, green and white, respectively. Right: the residues participating in hydrogen bonding in peptides. The receptor (LPS) and peptide chains of A6 or G6 are shown as green and blue, respectively. The hydrogen bonds are indicated as red dotted lines.

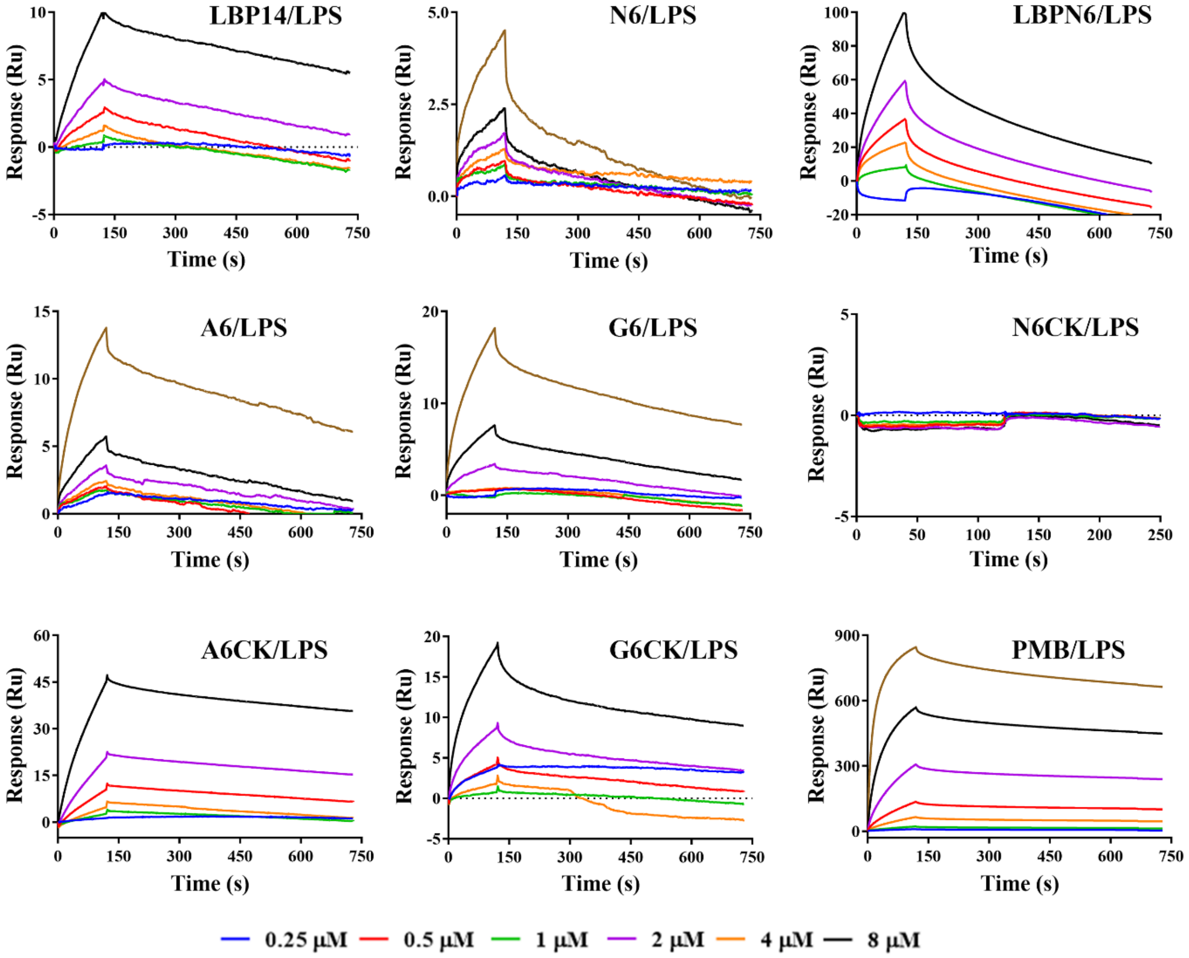

Supplementary Figure 11. SPR analysis of the interaction between peptides and immobilized ligands-LPS. Sensorgrams indicated the association and dissociation phases of the interactions between LPS and peptides or antibiotic (LBP14, N6, LBPN6, A6, G6, N6CK, A6CK, G6CK and PMB) at different concentrations (8, 4, 2, 1, 0.5, 0.25, and 0.125 mM) from top to bottom. The sensorgrams were fit with a 1:1 binding kinetic model.

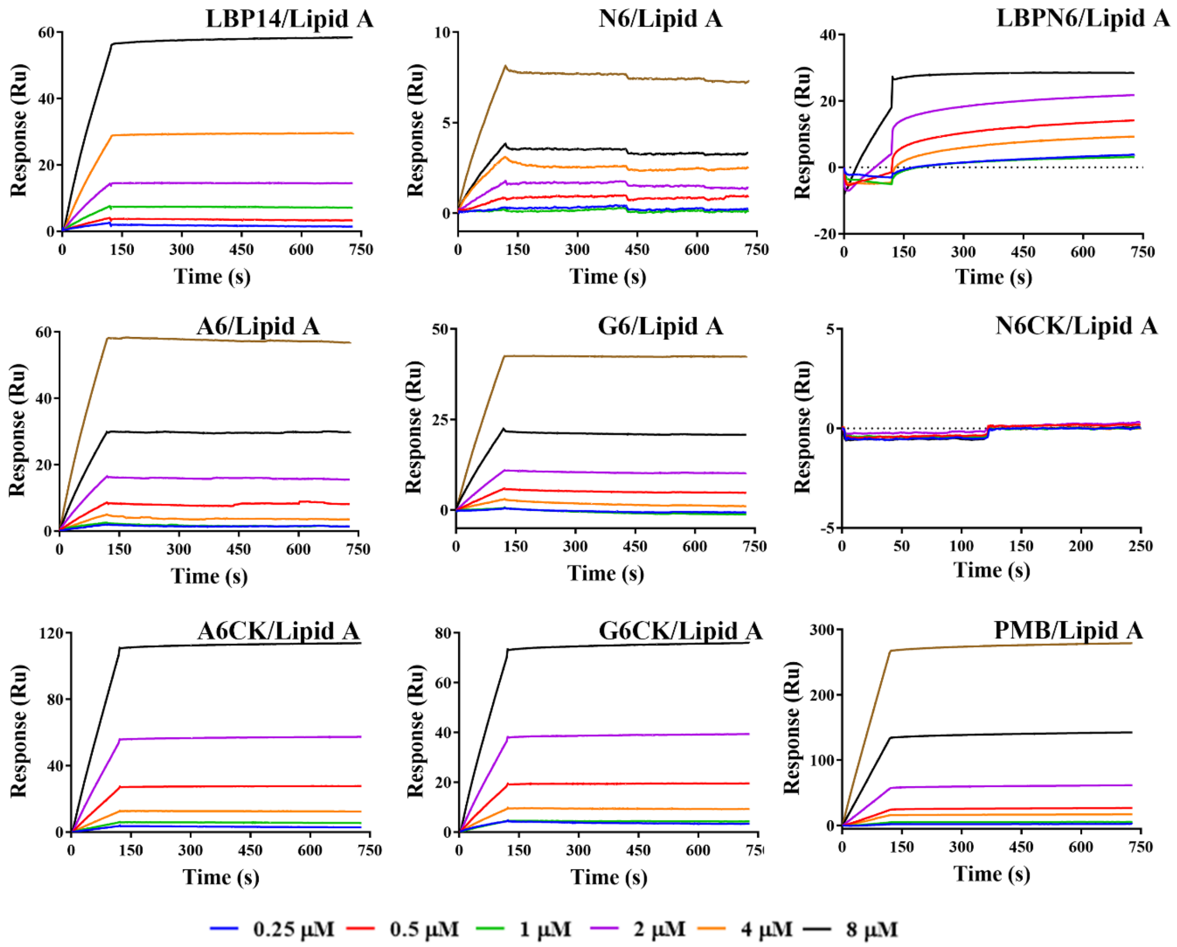

Supplementary Figure 12. SPR analysis of the interaction between peptides and immobilized ligands-lipid A. Sensorgrams indicated the association and dissociation phases of the interactions between lipid A and peptides or antibiotic (LBP14, N6, LBPN6, A6, G6, N6CK, A6CK, G6CK and PMB) at different concentrations (8, 4, 2, 1, 0.5, 0.25, and 0.125 mM) from top to bottom. The sensorgrams were fit with a 1:1 binding kinetic model.

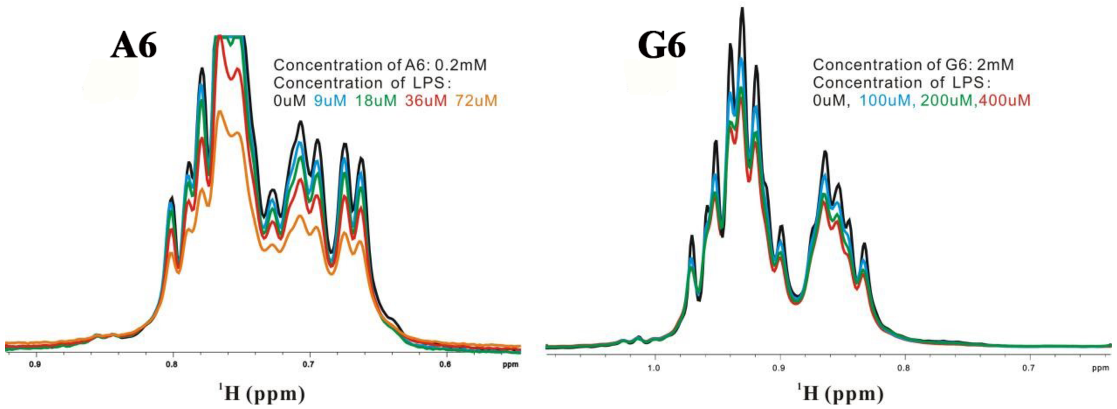

Supplementary Figure 13. Part of the proton NMR spectra for SCPs in presence of LPS. It indicated the line broadening effect on the adding of LPS to the peptide solvent.

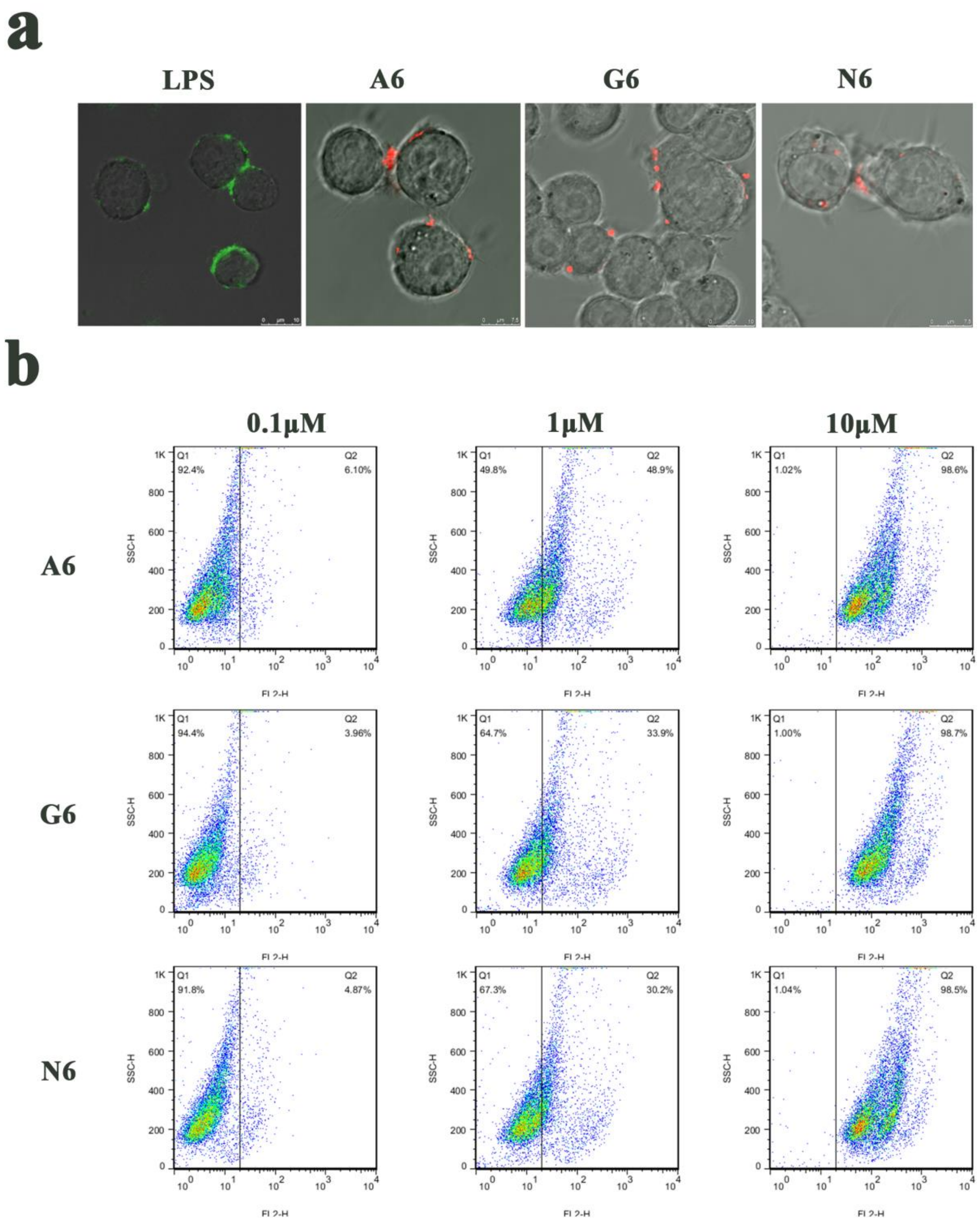

Supplementary Figure 14. SCPs binding to RAW 264.7 macrophages. Macrophage cells were treated with rhodamine-labeled peptides (0.1 µM) or FITC-labeled LPS (100 µg per ml) alone and analyzed by CLSM **(a)** and flow cytometry **(b)**. The scalebar represents 7.5 and 10 µm.

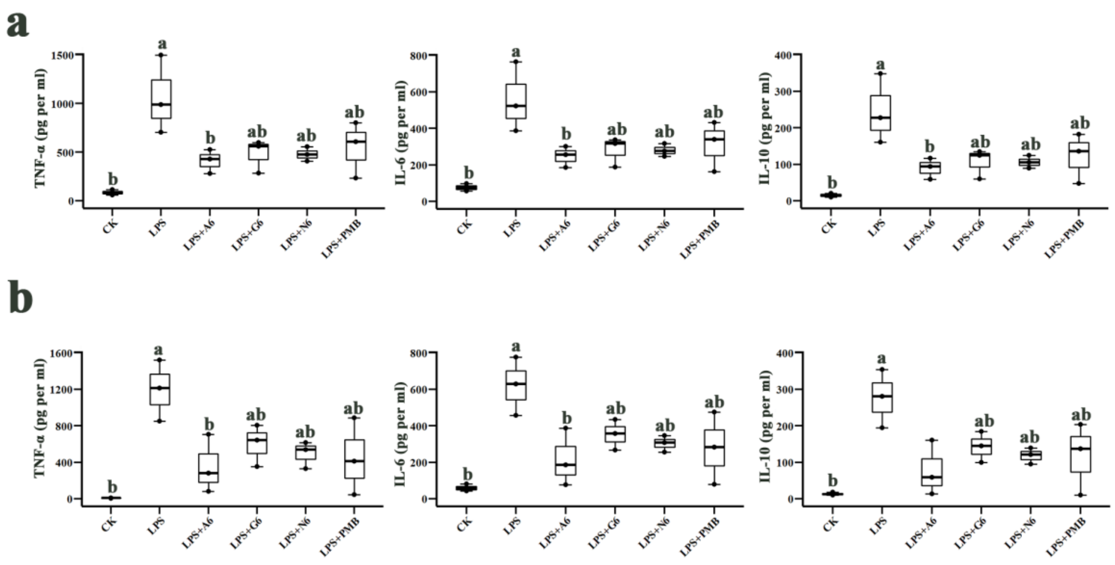

Supplementary Figure 15. Effects of SCPs on cytokines in RAW 264.7 ells. Cells were incubated with 0.1 μg per ml **(a)** or 1 μg per ml **(b)** LPS and 20 μM A6, G6, N6 or PMB for 2 h. The levels of TNF-α, IL-6 and IL-10 in supernatants were measured using ELISA. The data are given as the mean ± SD (n = 3 independent experiments). Different lower case letters indicate a significant difference between two groups ($p < 0.05$).

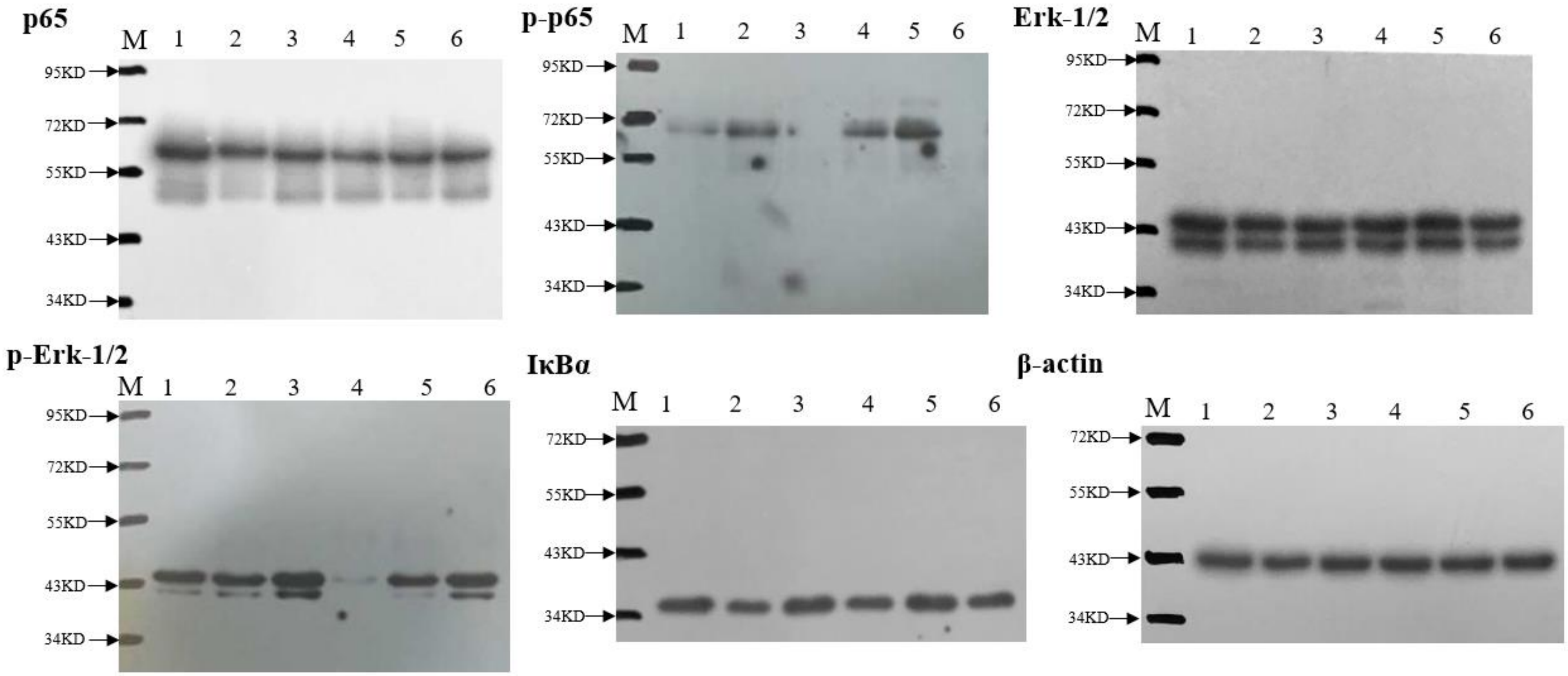

Supplementary Figure 16. A full and uncropped presentation for Fig. 7a. M: protein marker 26617 (Thermo Scientific™), Lane 1: CK, Lane 2: LPS, Lane 3: A6, Lane 4: G6, Lane 5: N6, Lane 6: PMB. Three samples from three biological repeats were homogenated and analyzed in a gel electrophoresis.

Supplementary Tables

Supplementary Table 1 Amino acid sequences and physicochemical properties of peptides.

Name	Sequence	MW (Da)	PI	Charge (+)	GRAVY	AI	AAS	BI (kcal per mol)	AH (%)[a]
LBP14	RVQGRWKVRASFFK	1765.10	12.31	5	-0.793	48.57	NN	NN	14.29
N6	GFAWNVCVYRNGVRVCHRRAN	2477.85	10.72	4	-0.310	64.76	0.229	2.61	0.00
A6	LBP14-(EA$_3$K)$_2$-N6	5165.99	11.33	9	-0.480	30.40	0.027	2.54	37.78
G6	LBP14-G$_4$S-N6	4540.22	11.89	9	-0.500	51.00	0.471	2.50	7.50
LBPN6	LBP14-N6	4224.93	11.89	9	-0.503	58.29	0.333	2.87	20
N6CK	VCVYRGFAWNCHRRANNGVRV	2477.85	10.72	4	-0.310	64.76	0.229	2.61	23.81
A6CK	LBP14-(EA$_3$K)$_2$-N6CK	5165.99	11.33	9	-0.480	58.67	0.027	2.54	64.44
G6CK	LBP14-G$_4$S-N6CK	4540.22	11.89	9	-0.500	51.00	0.471	2.50	20

MW: molecular weight; PI: isoelectric point; GRAVY: grand average of hydropathicity; NN: no data; AI: aliphatic index; AAS: antibacterial activity score; BI: Boman index; AH: alpha helix; a: calculated by NPS@; underlined residues: disulphide bond. LBP14N6 without linkers was used as a control; N6CK, A6CK and G6CK were designed as scramble controls of N6, A6, and G6, respectively.

Supplementary Table 2 CD analysis of secondary structures of SCPs and control peptides in the presence or absence of LPS.

Secondary structures	The percentages of secondary structures (%)															
	A6	A6+LPS	G6	G6+LPS	N6	N6+LPS	LBP14	LBP14+LPS	LBPN6	LBPN6+LPS	N6CK	N6CK+LPS	A6CK	A6CK+LPS	G6CK	G6CK+LPS
α-Helix	12.50	16.26	10.97	14.03	13.46	33.3	11.29	26.17	12.48	14.17	12.78	10.46	12.66	13.77	12.26	17.12
Antiparallel	35.45	23.22	35.85	24.39	46.94	1.73	40.25	12.03	24.72	20.03	42.85	45.20	33.48	30.82	40.45	27.30
Parallel	8.70	9.95	9.08	10.43	6.19	9.54	8.13	9.36	11.76	11.97	7.19	7.44	9.05	9.21	7.79	8.93
β-Turn	15.72	15.94	15.09	15.26	17.18	12.49	15.64	16.52	17.09	16.69	16.41	15.96	15.60	15.75	16.08	16.63
Random coil	27.63	34.63	29.00	35.97	16.23	42.93	24.68	35.91	34.02	37.13	20.70	20.86	29.21	30.44	23.43	30.02

Supplementary Table 3 Binding of SCPs and control peptides to LPS or lipid A in SPR.

Analytes	Ligands	kd (1 per Ms)	kd (1 per s)	KD (M)
LPS	A6	1.56×10^{3}	1.20×10^{-3}	7.68×10^{-7}
	G6	1.47×10^{3}	1.24×10^{-3}	8.46×10^{-7}
	N6	1.03×10^{3}	4.86×10^{-3}	4.71×10^{-6}
	PMB	6.66×10^{3}	3.02×10^{-4}	4.54×10^{-8}
	LBP14	1.91×10^{1}	1.32×10^{-3}	6.90×10^{-5}
	LBPN6	4.59×10^{2}	5.26×10^{-3}	1.15×10^{-5}
	N6CK	NN	NN	NN
	A6CK	1.87×10^{2}	4.84×10^{-4}	2.59×10^{-6}
	G6CK	1.42×10^{1}	1.43×10^{-3}	1.00×10^{-4}
Lipid A	A6	5.42×10^{2}	2.98×10^{-5}	5.49×10^{-8}
	G6	3.79×10^{2}	2.49×10^{-5}	6.57×10^{-8}
	N6	5.40×10^{2}	1.52×10^{-4}	2.81×10^{-7}
	PMB	7.08×10^{2}	4.49×10^{-7}	6.34×10^{-10}
	LBP14	7.03×10^{1}	1.01×10^{-7}	1.44×10^{-9}
	LBP-N6	6.90×10^{2}	4.14×10^{-7}	6.00×10^{-10}
	N6CK	NN	NN	NN
	A6CK	8.29×10^{1}	8.08×10^{-7}	9.75×10^{-9}
	G6CK	1.33×10^{2}	1.82×10^{-7}	1.39×10^{-9}

NN: no data; peptides could not bind with LPS or lipid A.

Supplementary Table 4 Total radiant efficiency of SCPs, N6 and free FITC in mice.

Time (h)	Free FITC	FITC-labeled N6	FITC-labeled A6	FITC-labeled G6	CK
0.5	1.03×10^{12}	1.18×10^{11}	1.87×10^{11}	1.90×10^{11}	3.08×10^{10}
1	1.63×10^{12}	1.36×10^{11}	1.48×10^{11}	1.55×10^{11}	2.89×10^{10}
2	1.52×10^{12}	1.13×10^{11}	7.09×10^{10}	7.41×10^{10}	2.90×10^{10}
4	1.11×10^{12}	1.23×10^{11}	6.71×10^{10}	8.14×10^{10}	4.44×10^{10}
8	3.35×10^{11}	6.64×10^{10}	4.49×10^{10}	5.28×10^{10}	3.81×10^{10}
24	2.22×10^{11}	6.26×10^{10}	4.74×10^{10}	6.27×10^{10}	4.90×10^{10}
48	1.17×10^{11}	3.75×10^{10}	3.07×10^{10}	4.05×10^{10}	3.25×10^{10}
72	9.36×10^{10}	3.72×10^{10}	3.01×10^{10}	4.06×10^{10}	3.37×10^{10}

Supplementary Notes

The *E. coli* CVCC195 strain is resistant to multiple antibiotics, including tetracyclines, lincosamindes, and aminoglycosides, making it very difficult to treat by antibiotics.

Supplementary Methods

Mixed-culture bactericidal kinetics of SCPs-A6 and G6. The mid-exponential phage *E. coli* CVCC195 and *S. aureus* ATCC43300 cultures were diluted to 2 × 10^5 CFU per ml, mixed (1:1) and added into the 96-well plates. A6, G6 or N6 were added into each well of plates to final concentration of 16 (A6 and G6) or 2 (N6) μg per ml in a 200-μl total volume. An aliquot was removed at 1, 3 and 5 min, respectively, and survivors were counted on MH solid plates[1–3].

MD. The backbones of ligand (A6 and G6) were kept rigid, and side chains were flexible. LPS was defined as a receptor and was kept rigid. Grid maps of LPS were set as 70 × 80 × 80, and the space is 0.375 Å, We define the H2 atom of LPS as the center. Docking of ligand with receptor was generated by using a Lamarchian genetic algorithm (LGA) with a translation step of 0.2 Å, a quaternion step of 5°, and a torsion step of 5°. The maximum number of energy evaluations increased to 15,000,000, 200 LGA docking were ran.

Effects of SCPs on inflammatory cytokines and IAP. The serum sample (50 μl) was added into each well of the plate; 50 μl of TNF-α, IL-I, IL-10 or IAP standard, control and test samples were then added to each well of the plate, followed by placing the latex film on the shaker and incubation for 2 h at room temperature. After discarding the liquid in the well, the enzyme labeling plate was placed in a 400 μl washing solution in the washing machine for 4 times; the enzyme labeling plate is inverted and tapped on a clean paper towel so that there was no residual liquid in each hole. Mouse TNF-α，IL-I，IL10 or IAP Conjugate (HRP Conjugate Detection Ab) working solution (100 μl) was added to each well of the plate (diluted 1:100 before the test); the latex film was placed on the shaker and incubated for 2 h at room temperature. After discarding the liquid in the well, the enzyme labeling plate was placed in a 400 μl washing solution in the washing machine for 4 times, then the enzyme

labeling plate is inverted, and tapped on a clean paper towel so that there is no residual liquid in each hole. Substrate Solution (100 µl) was added to each well of the plate and incubated at room temperature for 30 min in the dark. Finally, 100 µl of Stop Solution (2N sulfuric acid solution) was added to each well of the plate and gently mixed the plate to stop the reaction. The optical density value was read by placing the microplate in a microplate reader for 30 min with the reading wavelength of 450 nm and the calibration wavelength of 570 nm.

References:

1. Kaplan, C. W., et al. Selective membrane disruption: mode of action of C16G2, a specifically targeted antimicrobial peptide. *Antimicrob Agents Chemother.* **55**(7), 3446–3452 (2011).
2. Eckert, R., et al. Adding selectivity to antimicrobial peptides: rational design of a multidomain peptide against *Pseudomonas* spp. *Antimicrob Agents Chemother.* **50**(4), 1480–1488 (2006).
3. Eckert, R., et al. Targeted killing of *Streptococcus mutans* by a pheromone-guided "smart" antimicrobial peptide. *Antimicrob Agents Chemother.* **50**(11), 3651–3657 (2006).
