# Supplementary material for: Development of chimeric peptides to facilitate the neutralisation of lipopolysaccharides during bactericidal targeting of multidrug-resistant Escherichia coli
Source: Commun Biol. 2020 Jan 23;3:41. doi: 10.1038/s42003-020-0761-3 (PMC6978316; doi:10.1038/s42003-020-0761-3)
Supplement: Supplementary file 2 — Description of Additional Supplementary Files [file 42003_2020_761_MOESM2_ESM.docx]

Description of additional supplementary items

File Name: Supplementary Data 1

Description: Source data file

Fig. 2a Source data for the time-killing curves of A6, G6 and N6 against *E. coli* CVCC195 *in vitro*.

Fig. 2b Source data for effects of temperature on the antibacterial activity of A6, G6 and N6 against *E. coli* CVCC195.

Fig. 2c Source data for effects of pH on the antibacterial activity of A6, G6 and N6 against *E. coli* CVCC195.

Fig. 2d Source data for effects of enzyme on the antibacterial activity of A6, G6 and N6 against *E. coli* CVCC195.

Fig. 2e Source data for effects of serum on the antibacterial activity of A6, G6 and N6 against *E. coli* CVCC195.

Fig. 2f Source data for the peptide remaining in serum.

Fig. 2g Source data for resistance of A6 and G6.

Fig. 2h Source data for the hemolysis of A6, G6 and N6 against fresh mouse red blood cells.

Fig. 2i Source data for the cytotoxicity of A6, G6 and N6 against RAW 264.7 monocytes.

Fig. 4a Source data for dissociation of LPS aggregates.

Fig. 4b Source data for CD spectra for A6, G6 or N6 with or without *E. coli* LPS (0.2 mg per ml).

Fig. 4c Source data for binding affinity of LBP14, A6, G6 and N6 to LPS.

Fig. 5a Source data for effects of A6 and G6 on the binding of LPS and LBP.

Fig. 6a Source data for survival of mice.

Fig. 6b Source data for effects of A6 and G6 on the cytokines and IAP levels.

Fig. 7b Source data for densitometric analysis of p-p65/p65 ratio.

Fig. 7c Source data for densitometric analysis of p-ERK1/2/ERK1/2 ratio.

Fig. 7d Source data for densitometric analysis of IκBα/β-actin ratio.

Supplementary Fig. 4 Source data for killing selectivity of peptides.

Supplementary Fig. 6a Source data for CD spectra for LBP with or without *E. coli* LPS (0.2 mg per ml).

Supplementary Fig. 6b Source data for CD spectra for N6CK with or without *E. coli* LPS (0.2 mg per ml).

Supplementary Fig. 6c Source data for CD spectra for LBPN6 with or without *E. coli* LPS (0.2 mg per ml).

Supplementary Fig. 6d Source data for CD spectra for A6CK with or without *E. coli* LPS (0.2 mg per ml).

Supplementary Fig. 6e Source data for CD spectra for G6CK with or without *E. coli* LPS (0.2 mg per ml).

Supplementary Fig. 6f Source data for CD spectra for LPS with or without *E. coli* LPS (0.2 mg per ml).

Supplementary Fig. 13a Source data for effects of SCPs on cytokines in RAW 264.7 ells incubated with 0.1 μg per ml LPS.

Supplementary Fig. 13b Source data for effects of SCPs on cytokines in RAW 264.7 ells incubated with 1 μg per ml LPS.
